# The primary motor cortex of schizophrenia patients show neuronal and subcellular impairments in the right hemisphere – postmortem study

**DOI:** 10.1192/j.eurpsy.2024.779

**Published:** 2024-08-27

**Authors:** P. Szocsics, P. Papp, L. Havas, J. Lőke, Z. Maglóczky

**Affiliations:** ^1^Human Brain Research Laboratory, HUN-REN, Institute of Experimental Medicine; ^2^János Szentágothai Doctoral School of Neuroscience, Semmelweis University; ^3^Cerebral COrtex Research Group, HUN-REN, Institute of Experimental Medicine, Budapest; ^4^Department of Pathology; ^5^Department of Psychiatry, St. Borbála Hospital, Tatabánya, Hungary

## Abstract

**Introduction:**

In mental disorders, very little is known about the cellular and subcellular mechanisms underlying the development of symptoms. Postmortem studies can contribute to understanding these. Our research group collects and studies cortical samples with short postmortem intervals from schizophrenia patients.

**Objectives:**

We investigated primary motor cortical brain samples, to understand the background of motor symptoms in schizophrenia.

**Methods:**

Both hemispheres of primary motor cortices of eight control- and eight subjects with schizophrenia were analysed by immunohistochemistry. We labelled pyramidal cells with SMI32 antibody, which binds to neurofilaments, and parvalbumin (PV) antibody, which labels one type of inhibitory input on these cells, axo-axonic and axo-somatic interneurons, and a proportion of giant pyramidal neurons (Betz cells). We were interested in the size and density of layer 3 and 5 pyramidal cells and Betz cells, the distribution of PV-labelled terminals and the PV expression of Betz cells. Results of the subjects were compared both as a whole and separately per hemisphere.

**Results:**

Most changes were present in the primary motor cortices in the right hemisphere (presumably subdominant). Here, the density of Betz cells and their inhibitory inputs were also reduced. PV-expression of Betz cells was not dependent on the group studied, but we observed that it is decreasing with age. The other investigated characteristics show no significant differences.

**Conclusions:**

Our results suggest that the primary motor cortex may be involved in schizophrenia. Neurodevelopmental, pharmacological and neurodegenerative causes could be involved in this process. Network dysconnectivity is likely to underlie the stronger involvement of the subdominant side, and literature data point also in this direction. We believe that our research method is suitable for the study of the background of other symptoms and may lead to a better understanding of schizophrenia, especially if we could combine our results with clinical research.

**Disclosure of Interest:**

None Declared

